# FRET score: predictors of futile recanalisation following endovascular thrombectomy—a multicentre cohort study from the EVATRISP collaboration

**DOI:** 10.1093/esj/aakaf013

**Published:** 2026-01-01

**Authors:** Yoel Schwartzmann, Mirjam R Heldner, Hamza Jubran, Marcel Arnold, Philipe S Breiding, Fatma Shalabi, Tamer Jubeh, Issa Metanis, Annika Nordanstig, Paul J Nederkoorn, Nabila Wali, Anne van der Meij, Susanne Wegener, Lukas Otto, Hannah Lea Handelsmann, Patrik Michel, Davide Strambo, Alexander Salerno, Gian Marco De Marchis, Tolga Dittrich, Sami Curtze, Nicolas Martinez-Majander, Henrik Gensicke, Stefan Engelter, Valerian Altersberger, Simon Trüssel, Christian H Nolte, Christoph Riegler, Andrea Zini, Federica Naldi, Guido Bigliardi, Livio Picchetto, Joao Pedro Marto, José Pedro Costa, Jeremy Molad, Hen Hallevi, Carlo W Cereda, Alessandro Pezzini, Mauro Magoni, Visnja Padjen, Marialuisa Zedde, Ronen R Leker

**Affiliations:** Department of Neurology, Hadassah-Hebrew University Medical Center, Jerusalem, Israel; Department of Neurology, Inselspital, Bern University Hospital and University of Bern, Bern, Switzerland; Department of Neurology, Hadassah-Hebrew University Medical Center, Jerusalem, Israel; Department of Neurology, Inselspital, Bern University Hospital and University of Bern, Bern, Switzerland; University Institute of Diagnostic and Interventional Neuroradiology, Inselspital, Bern University Hospital and University of Bern, Bern, Switzerland; Department of Neurology, Hadassah-Hebrew University Medical Center, Jerusalem, Israel; Department of Neurology, Hadassah-Hebrew University Medical Center, Jerusalem, Israel; Department of Neurology, Hadassah-Hebrew University Medical Center, Jerusalem, Israel; Department of Neurology, Sahlgrenska University Hospital, Gothenburg, Sweden; Department of Neurology, Amsterdam UMC, Location AMC, University of Amsterdam, Amsterdam, The Netherlands; Department of Neurology, Amsterdam UMC, Location AMC, University of Amsterdam, Amsterdam, The Netherlands; Department of Neurology, Amsterdam UMC, Location AMC, University of Amsterdam, Amsterdam, The Netherlands; Department of Neurology, University Hospital Zurich (USZ) and University of Zurich (UZH), Zurich, Switzerland; Department of Neurology, University Hospital Zurich (USZ) and University of Zurich (UZH), Zurich, Switzerland; Department of Neurology, University Hospital Zurich (USZ) and University of Zurich (UZH), Zurich, Switzerland; Stroke Center, Neurology Service, Centre Hospitalier Universitaire Vaudois and University of Lausanne, Lausanne, Switzerland; Stroke Center, Neurology Service, Centre Hospitalier Universitaire Vaudois and University of Lausanne, Lausanne, Switzerland; Stroke Center, Neurology Service, Centre Hospitalier Universitaire Vaudois and University of Lausanne, Lausanne, Switzerland; Department of Neurology, University Teaching and Research Hospital, HOCH-Kantonsspital St. Gallen, St. Gallen, Switzerland; Medical Faculty, University of Basel, Basel, Switzerland; Department of Neurology, University Teaching and Research Hospital, HOCH-Kantonsspital St. Gallen, St. Gallen, Switzerland; Medical Faculty, University of Basel, Basel, Switzerland; Department of Neurology, Helsinki University Hospital and Clinical Neurosciences, University of Helsinki, Helsinki, Finland; Department of Neurology, Helsinki University Hospital and Clinical Neurosciences, University of Helsinki, Helsinki, Finland; Department of Rehabilitation and Neurology, University Department of Geriatric Medicine FELIX PLATTER, University of Basel, Basel, Switzerland; Department of Neurology and Stroke Center, University of Basel, Basel, Switzerland; Department of Clinical Research, University of Basel, Basel Switzerland; Department of Rehabilitation and Neurology, University Department of Geriatric Medicine FELIX PLATTER, University of Basel, Basel, Switzerland; Department of Neurology and Stroke Center, University of Basel, Basel, Switzerland; Department of Clinical Research, University of Basel, Basel Switzerland; Department of Rehabilitation and Neurology, University Department of Geriatric Medicine FELIX PLATTER, University of Basel, Basel, Switzerland; Department of Neurology and Stroke Center, University of Basel, Basel, Switzerland; Department of Clinical Research, University of Basel, Basel Switzerland; Department of Rehabilitation and Neurology, University Department of Geriatric Medicine FELIX PLATTER, University of Basel, Basel, Switzerland; Department of Neurology and Stroke Center, University of Basel, Basel, Switzerland; Department of Clinical Research, University of Basel, Basel Switzerland; Department of Neurology with experimental Neurology, Charité Universitätsmedizin Berlin, Berlin, Germany; Center for Stroke Research Berlin (CSB), Charité Universitätsmedizin Berlin, Berlin, Germany; Berlin Institute for Health (BIH), Charité Universitätsmedizin Berlin, Berlin, Germany; Department of Neurology with experimental Neurology, Charité Universitätsmedizin Berlin, Berlin, Germany; Center for Stroke Research Berlin (CSB), Charité Universitätsmedizin Berlin, Berlin, Germany; Berlin Institute for Health (BIH), Charité Universitätsmedizin Berlin, Berlin, Germany; Department of Neurology and Stroke Center, Maggiore Hospital, IRCCS Istituto Delle Scienze Neurologiche di Bologna, Bologna, Italy; Department of Neurology and Stroke Center, Maggiore Hospital, IRCCS Istituto Delle Scienze Neurologiche di Bologna, Bologna, Italy; Neurology-Stroke Unit, Ospedale Civlie di Baggiovara, Azienda Ospedaliero Universitaria di Modena, Modena, Italy; Neurology-Stroke Unit, Ospedale Civlie di Baggiovara, Azienda Ospedaliero Universitaria di Modena, Modena, Italy; Lisbon Clinical Academic Center, NOVA Medical School, Universidade NOVA de Lisboa, Lisbon, Portugal; Lisbon Clinical Academic Center, NOVA Medical School, Universidade NOVA de Lisboa, Lisbon, Portugal; Department of Stroke & Neurology, Tel-Aviv Sourasky Medical Center, Tel-Aviv, Israel; Faculty of Medicine, Tel-Aviv University, Tel-Aviv, Israel; Department of Stroke & Neurology, Tel-Aviv Sourasky Medical Center, Tel-Aviv, Israel; Faculty of Medicine, Tel-Aviv University, Tel-Aviv, Israel; Stroke Center EOC, Neurocenter of Southern Switzerland, Ospedale Regionale di Lugano, Ente Ospedaliero Cantonale, Lugano, Switzerland; Department of Medicine and Surgery, University of Parma, Parma, Italy; Stroke Care Program, Department of Emergency, Parma University Hospital, Parma, Italy; USD Stroke Unit and Vascular Neurology, ASST Spedali Civili di Brescia, Brescia, Italy; Neurology Clinic, University Clinical Centre of Serbia, Faculty of Medicine, University of Belgrade, Belgrade, Serbia; Neurology Unit-Stroke Unit, Azienda Unità Sanitaria Locale-IRCCS di Reggio Emilia, Reggio Emilia, Italy; Department of Neurology, Hadassah-Hebrew University Medical Center, Jerusalem, Israel

**Keywords:** endovascular treatment, futile recanalisation, large vessel occlusion, stroke

## Abstract

**Introduction:**

Endovascular thrombectomy (EVT) is the treatment of choice for LVO stroke, yet nearly half of successfully recanalised patients fail to achieve functional independence, a phenomenon termed futile recanalisation (FR). Predictors of FR remain poorly defined in large, heterogeneous populations. Therefore, we aimed to develop a predictive score for FR.

**Patients and methods:**

Endovascular thrombectomy-treated LVO patients from the prospective, multicentre EVATRISP collaboration were included. All patients had known pre-stroke functional status, modified thrombolysis in cerebral infarction (mTICI) score and 90-day mRS. Futile recanalisation was defined as mRS > 2 at 90 days despite mTICI ≥ 2b. Patients with FR were compared to those with successful recanalisation and mRS ≤ 2. The cohort was randomly split into derivation (70%) and validation (30%) sets. Multivariable logistic regression identified independent predictors that were used to construct the futile recanalisation following endovascular thrombectomy (FRET) score.

**Results:**

Of 9909 patients, 7272 (73%) achieved successful recanalisation and 3420 (47%) of them experienced FR. In the derivation set, FR was independently associated with older age, diabetes, ischaemic heart disease, higher NIHSS, anterior cerebral artery occlusion, seizures at presentation, non-use of intravenous thrombolysis and lower Alberta Stroke Program Early CT Score (ASPECTS) or posterior circulation ASPECTS. Futile recanalisation patients had longer hospital stays and higher mortality rates. The FRET score demonstrated good discrimination (area under the curve [AUC] 0.721; 95% CI, 0.702–0.740), with FRET ≥ 3 indicating high risk. The validation cohort yielded similar performance (AUC 0.708; 95% CI, 0.680–0.737).

**Conclusion:**

The FRET score enables early identification of EVT patients at high risk for FR.

## Graphical Abstract

**Graphical Abstract:**
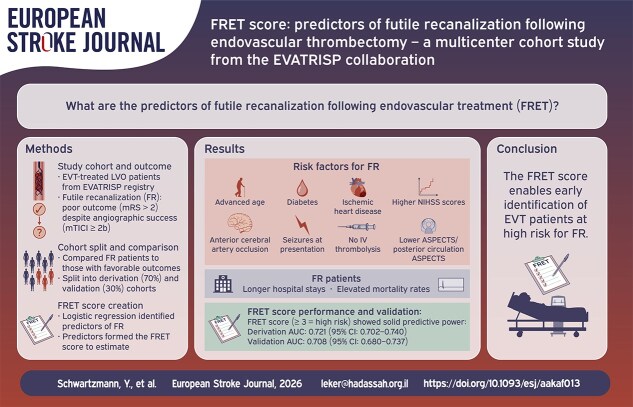

## Introduction

Endovascular thrombectomy (EVT) has proven superior to best medical treatment in patients with LVO stroke.^1–4^

Despite the beneficial effects of EVT, the rates of functional independence following EVT remain considerably low (14%–46%) and rates of bedridden state or death are up to 50%.^2^^,^^3^^,^^5^^,^^6^

Successful recanalisation, defined as modified thrombolysis in cerebral infarction (mTICI) score ≥ 2b, can be achieved in 73%–88% of LVO patients.^1^^,^^3^^,^^4^^,^^7^ Despite successful recanalisation, patients may still experience unfavourable functional outcome, a constellation termed futile recanalisation (FR). Futile recanalisation has a prevalence of 40%–51% and previous studies found it to be associated with several risk factors.^8–11^ Predictive models have been developed to assess the risk of FR, including the BAND (Baseline mRS, age, NIHSS, delay from last known normal), SNAP (Site of occlusion, NIHSS, age, pre-stroke mRS) and PANDA (Pre-stroke disability, age, NIHSS, delay from last known normal, and ASPECTS) scores.^8–13^ However, these models are limited by small sample sizes, single-centre designs and heterogeneous patient selection criteria. Furthermore, these studies only included patients with anterior circulation infarcts. These limitations raise concerns regarding the generalisability and overall quality of the data.

Endovascular thrombectomy is often costly and labour-intensive, and performance of EVT in patients with a poor prognosis could lead to increased costs without clear benefit. Therefore, identifying factors associated with FR could improve patient selection for EVT. This approach could potentially result in significant cost savings and reduce the burden on high-volume EVT centres.

We aimed to identify risk factors associated with FR in a large cohort from an international, prospective multi-centre registry, the Endovascular treatment and Thrombolysis for Ischaemic Stroke Patients (EVATRISP) collaboration in order to develop a simple all-inclusive predictive tool for FR.

## Patients and methods

Endovascular Treatment and Thrombolysis for Ischaemic Stroke Patients is an international, multi-centre cohort study enrolling consecutive patients receiving EVT. Detailed information about the EVATRISP study protocol has previously been published.^14^ The institutional ethics review boards in each individual participating centre approved the study, with a waiver of informed consent due to the observational design, the anonymised nature of data collection and the retrospective approach in some centres, while in others, informed consent was obtained if not waived by the respective authorities in participating centres.

For the current analysis, patients with FR were compared to those without FR.

Inclusion criteria for this analysis included EVT performed during 2015–2024, age ≥ 18 years and LVO occlusion, diagnosed by CTA, MRA or digital subtraction angiography. In addition, patients were required to have evidence of successful recanalisation, defined as mTICI scores ≥ 2b following EVT. Of note, absolute TICI scores were not registered in the EVATRISP database, and EVT results were only classified as successful vs unsuccessful with the agreement that successful recanalisation is TICI ≥ 2.

Exclusion criteria included pre-stroke disability, defined as mRS > 2, missing data on pre-stroke or 90 days post-stroke mRS and unknown recanalisation status following EVT.

Predefined variables were collected at 18 comprehensive stroke centres from 9 different countries, as previously described.^14^ Briefly, collected data included demographics, risk factor profiles, clinical findings, baseline imaging characteristics including non-contrast CT with ASPECTS or posterior circulation ASPECTS (pcASPECTS) when appropriate, CTA with collateral status assessed using the Tan score, medical treatment administered and clinical and radiological outcomes following intervention.^15–17^

Statistical analyses were performed using SPSS Statistics version 29 (IBM Corporation, Armonk, NY, USA). The cohort was randomly divided into a derivation (70%) and validation (30%) sets. In the derivation set, we compared favourable functional outcome (mRS ≤ 2) to non-favourable functional outcome (mRS > 2) at day 90 post stroke. We also performed a sensitivity analysis looking at outcomes of mRS > 3 as FR.

A *P*-value < .05 was considered significant. The chi-square (χ^2^) test was used to evaluate associations between categorical variables. Student’s *t*-test and Fisher’s exact test were applied for comparisons of continuous parametric and small-sample categorical variables. For nonparametric comparisons, the median test with IQR was used. Correlations between variables were assessed using the Pearson correlation coefficient and we also performed tests for collinearity of variables.

We next performed multivariable logistic regression modelling to identify variables associated with the likelihood of FR. Pre-EVT factors with *P* < .05 in univariable analysis were entered. A second multivariable regression model was next performed that included variables known to affect outcomes including age, pre-stroke mRS, NIHSS on admission, time to groin puncture and ASPECT score.

To develop a predictive model for FR, we constructed a clinical scoring system based on variables that were independently associated with FR in the multivariate logistic regression analysis. Variables with sufficient data availability were included, and each was assigned points according to its relative effect size.

To evaluate the discriminative performance of the score, a receiver operating characteristic (ROC) curve was generated in the derivation set using FR as the outcome, and the area under the curve (AUC) was calculated. The optimal cutoff point was determined using Youden’s Index. The results were further compared to existing predictive methods that were already published including the BAND, SNAP and PANDA scores.^8–13^

For internal validation, the model was applied to a validation cohort to assess the score’s predictive performance in an independent subset.

## Results

Of the 15,549 patients included in the EVATRISP registry, 9909 had LVO with a pre-stroke mRS ≤ 2, available data on 90-day mRS and known recanalisation status (Figure S1). There were some minor differences in baseline characteristics between included and excluded patients (Table S1).

Of the 9909 patients with available data, 7272 (73%) achieved successful recanalisation (Figure S1). Patients with successful recanalisation were then randomly divided into a derivation (70%) and validation (30%) sets.

In the derivation set, 2371 (47%) patients experienced FR post EVT. Compared to those with favourable outcomes, patients with FR were older and less likely to be male (*P* < .001, for both). As shown in Table 1, vascular risk factors including atrial fibrillation, diabetes mellitus, arterial hypertension, smoking, prior stroke and coronary heart disease were more prevalent in the FR group (*P* < .001 for all). Epileptic seizures at stroke onset were more frequent in the FR group (*P* = .001). Patients in the FR group had higher admission NIHSS scores (*P* < .001), and higher systolic blood pressure at presentation (*P* = .021). Stroke etiology classified according to the Trial of ORG 10172 in Acute Stroke Treatment (TOAST) criteria, demonstrated statistically significant differences between groups (*P* = .004).

**Table 1:** Derivation cohort—demographic, comorbidities and clinical characteristic of patients with successful recanalisation following endovascular thrombectomy according to functional outcome at day 90

**Variable**	**mRS ≤ 2 (*n* = 2,701)**	**mRS > 2 (*n* = 2,371)**	***P*-value**
Sex male (%)	1,520 (56.3)	1,214 (51.2)	<.001
Age (median, IQR)	70 (59–78)	78 (69–84)	<.001
Transferred from another hospital (%)	902 (35.3)	811 (36.1)	.605
Atrial fibrillation (%)	846 (31.1)	975 (41.4)	<.001
Diabetes mellitus (%)	390 (14.4)	544 (22.9)	<.001
Hypertension (%)	1,674 (62.0)	1,743 (73.5)	<.001
Dyslipidemia (%)	1,358 (50.3)	1,196 (50.4)	.907
Smoking (%)	685 (25.4)	390 (16.4)	<.001
Coronary heart disease (%)	410 (15.2)	478 (20.2)	<.001
Prior ischaemic stroke (%)	271 (10.0)	335 (14.1)	<.001
Wake up stroke (%)	436 (16.1)	364 (15.4)	.441
Epileptic seizure at presentation (%)	18 (0.7)	38 (1.6)	.001
NIHSS score at presentation (median, IQR)	11 (6–17)	17 (12–21)	<.001
Systolic blood pressure at presentation mmHg (mean ± SD)	148 ± 29	150 ± 33	.021
Diastolic blood pressure at presentation mmHg (mean ± SD)	82 ± 18	82 ± 21	.798
TOAST criteria			.004
Large vessel atherosclerosis (%)	19.6	19.2	
Cardiac source (%)	43.8	46.9	
More than one etiology (%)	5.7	7.0	
Undetermined (%)	23.6	18.9	
Other (%)	7.2	8.1	

Abbreviation: TOAST = Trial of ORG 10172 in Acute Stroke Treatment.

Imaging characteristics prior to EVT, showed significantly lower ASPECT/pcASPECT scores in FR patients as well as more ischaemic changes, and poorer collaterals (*P* < .001 for all, Table 2).

**Table 2:** Derivation cohort—baseline radiological findings of patients with successful recanalisation following endovascular thrombectomy according to functional outcome at day 90

**Variable**	**mRS ≤ 2 (*n* = 2,701)**	**mRS > 2 (*n* = 2,371)**	***P*-value**
ASPECT/pcASPECTS score (median, IQR) (^*^n = 3,123)	9 (8–10)	9 (7–10)	<.001
Early ischaemic changes on computed tomography (%)	739 (27.4)	807 (34.0)	<.001
Tan score in M1/2 occlusion (median, IQR) (^*^n = 1,062)	2 (1–3)	2 (1–2)	<.001
Vessel occlusion			
Internal carotid (%)	173 (6.4)	171 (7.2)	.254
Terminus carotid (%)	279 (10.3)	377 (15.9)	.001
Middle cerebral artery proximal M1 (%)	907 (33.6)	804 (22.9)	.804
Middle cerebral artery distal M1 (%)	433 (16.0)	317 (13.4)	.008
Middle cerebral artery M2 (%)	680 (25.2)	482 (20.3)	<.001
Anterior cerebral artery (%)	27 (1.0)	49 (2.1)	.002
Posterior cerebral artery (%)	86 (3.2)	67 (2.8)	.457
Basilar artery (%)	146 (5.4)	168 (7.1)	.013
Vertebral artery (%)	39 (1.4)	39 (1.6)	.562

Abbreviations: ASPECTS = Alberta Stroke Program Early CT Score; pcASPECTS = posterior circulation ASPECTS.

The FR group exhibited a higher frequency of occlusions in the internal carotid artery terminus segment (*P* = .001), anterior cerebral artery (ACA) (*P* = .002) and basilar artery (*P* = .013). Conversely, the non-FR group had a greater proportion of occlusions in the distal MCA segments, including distal M1 (*P* = .008) and proximal dominant M2 segment (*P* < .001, Table 2).

Intravenous thrombolysis (IVT) was administered more frequently in the non-FR group (*P* < .001). Time from symptoms onset to recanalisation was significantly longer in the non-FR group (*P* = .018). However, time intervals from symptom onset to IVT administration, and groin puncture were comparable between groups (Table 3).

**Table 3:** Derivation cohort—medical treatment and clinical outcomes of patients with successful recanalisation following endovascular thrombectomy according to functional outcome at day 90

**Variable**	**mRS ≤ 2 (*n* = 2,701)**	**mRS > 2 (*n* = 2,371)**	***P*-value**
Symptoms onset to IVT, minutes (mean ± SD)	125 ± 188	123 ± 90	.905
Symptoms onset to groin puncture, minutes (mean ± SD)	266 ± 443	266 ± 303	.996
Symptom onset to recanalisation, minutes (mean ± SD)	719 ± 1,443	586 ± 1,252	.018
Tissue plasminogen activator (%)	1,092 (40.4)	685 (28.9)	<.001
Intra-arterial thrombolytic agent (%)	125 (4.6)	103 (4.3)	.627
General anaesthesia (%)	1,401 (51.9)	1,376 (58.0)	<.001
Number of passes (median, IQR) (^*^n = 2,369)	1 (1–2)	1(1–3)	<.001
Endovascular thrombectomy complication (%)	279 (10.3)	333 (14.0)	<.001
Any intracranial haemorrhage post procedure (%)	469 (17.4)	794 (33.5)	<.001
Symptomatic intracranial haemorrhage (%)	18 (0.7)	165 (7.0)	<.001
Subarachnoid or subdural haemorrhage post-EVT (%)	36 (1.3)	69 (2.9)	<.001
NIHSS score 24 hours (median, IQR)	3 (1–6)	14 (7–19)	<.001
mRS 3 months after thrombectomy (median, IQR)	1 (0–2)	4 (3–6)	<.001
Mortality day 90 (%)	15 (0.6)	877 (37.0)	<.001
Length of hospital stay, days (median, IQR)	5 (3–8)	6 (3–12)	<.001

Abbreviations: EVT = endovascular thrombectomy; IVT = intravenous thrombolysis.

The FR group more frequently underwent general anaesthesia, had a higher number of passes, and experienced higher complication rates related to the procedure (*P* < .001 for all, Table 3).

The incidence of post-procedural intracranial haemorrhages was higher in the FR group (*P* < .001 for all, Table 3).

NIHSS scores at 24 hours post-procedure were higher in the FR group as well as length of hospital stay and mortality rates at 90 days (*P* < .001 for all, Table 3).

Similar results were seen in the sensitivity analysis looking at mRS ≥ 3 as FR (5074 patients, Tables S2 and S3) with the only difference in predictors being that ACA involvement no longer remained a statistically significant predictor of FR.

We also performed a test for co-linearity of variables associated with outcome. Variance inflation factor values ranged between 1.0 and 1.4, indicating absence of multicollinearity.

We next performed a multivariable regression analysis (Table 4). Factors independently associated with FR included older age, diabetes mellitus, coronary heart disease, epileptic seizure at presentation, higher NIHSS score at presentation and occlusion of ACA.

**Table 4:** Regression analysis for presence of FR at day 90 post-stroke, for patients with successful recanalisation following EVT

	**Adjusted OR**	**95% CI**	***P*-value**
Sex male	1.12	0.94–1.34	.198
Age (per year)	1.04	1.03–1.05	<.001
Atrial fibrillation	0.99	0.82–1.19	.890
Diabetes mellitus	1.58	1.27–1.97	<.001
Hypertension	1.08	0.87–1.33	.475
Smoking	0.95	0.75–1.19	.637
Coronary heart disease	1.27	1.00–1.61	.047
Prior ischaemic stroke	1.09	0.83–1.44	.530
Seizure at stroke	3.66	1.38–9.65	.009
Admission NIHSS (per 1 point)	1.11	1.09–1.12	<.001
Admission systolic blood pressure (per mmHg)	1.00	0.99–1.01	.128
Tissue plasminogen activator	0.61	0.51–0.74	<.001
ASPECTS/pcASPECTS	0.87	0.83–0.92	<.001
Early ischaemic changes on CT	1.09	0.89–1.34	.382
Collateral status			
Tan score 1 vs 0	0.85	0.47–1.56	.62
Tan score 2 vs 0	0.57	0.32–1.04	.66
Tan score 3 vs 0	0.70	0.56–1.74	.27
Vessel involved			
Terminus carotid	1.14	0.87–1.45	.351
Middle cerebral artery distal M1	0.79	0.62–1.00	.055
Anterior cerebral artery	1.07	0.86–1.32	.534
Middle cerebral artery M2	2.01	1.05–3.81	.034
Basilar artery	1.06	0.56–2.02	.844
General anaesthesia	1.08	0.90–1.30	.377

Abbreviations: ASPECTS = Alberta Stroke Program Early CT Score; pcASPECTS = posterior circulation ASPECTS; TOAST = Trial of ORG 10172 in Acute Stroke Treatment.

Factors independently protecting from FR included use of IVT and higher ASPECT/pcASPECT score. A second regression model based on factors that were previously published to affect outcomes in patients with LVO confirmed that age, pre-stroke mRS, admission NIHSS, ASPECTS scores and time from symptom onset to EVT were all indeed associated with outcomes (Table S4).

We next constructed a predictive model for FR. The selected variables included: age > 75 years (1 point), age > 90 years (2 points); presence of diabetes, ischaemic heart disease, epileptic seizure, occlusion of ACA and non-use of tPA (1 point each); NIHSS > 17 (1 point) and ≥ 25 (2 points); ASPECTS/pcASPECTS 4–7 (1 point) and < 4 (2 points, Table 5). Specifically for ASPECTS/pcASPECTS scores we used a pragmatic approach based on the existing literature in patients with large core LVO showing that ASPECTS ≤ 3 is associated with poor outcomes despite EVT^18^ and from studies with small core LVO showing that ASPECTS ≥ 7 is associated with favourable outcomes.^2^

**Table 5:** The FRET score

**Predictor**	**Criteria**	**Points**
Age	>75	1
	>90	2
Diabetes mellitus	Present	1
Ischaemic heart disease	Present	1
Epileptic seizure at presentation	Present	1
NIHSS	17–24	1
	≥25	2
Anterior cerebral artery occlusion	Present	1
Tissue plasminogen activator	Absent	1
ASPECTS/pcASPECTS	4–7	1
	<4	2

Abbreviations: ASPECTS = Alberta Stroke Program Early CT Score; FRET = futile recanalisation following endovascular thrombectomy; pcASPECTS = posterior circulation ASPECTS.

**Figure 1:**
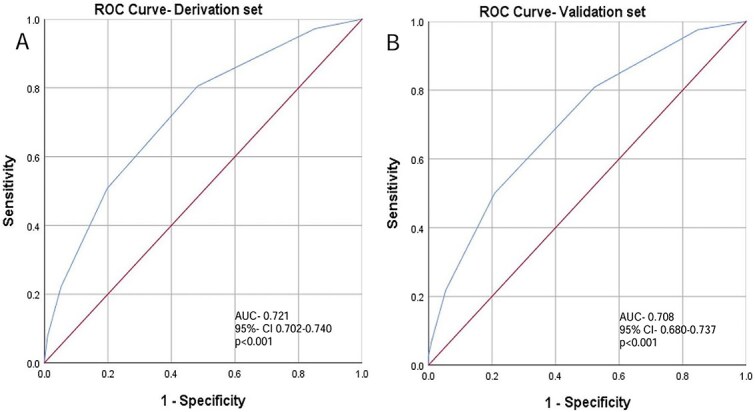
ROC curves of derivation (panel 1A) and validation sets (panel 1B). Abbreviations: AUC = area under the curve; CI = confidence interval; ROC = receiver operating characteristic.

The predictive performance of the score was evaluated using ROC curve analysis (Figure 1A). The predictive score demonstrated good discriminating ability with an AUC of 0.721 (95% CI, 0.702–0.740; *P* < .001). The optimal cutoff was 2.5. As the score is based on whole numbers value, a threshold of ≥ 3 was selected, which yielded a sensitivity of 50.1% and a specificity of 80.2% for predicting FR, with a positive predictive value (PPV) of 67.4%, negative predicative value (NPV) of 66.3%, positive likelihood ratio (LR^+^) of 2.53 and negative likelihood ratio (LR^−^) of 0.62.

A comparison with other published predictive models (Figure S2) showed that the AUC of futile recanalisation following endovascular thrombectomy (FRET) was similar to that obtained in the PANDA, BAND and SNAP models.

We next performed internal validation using the validation cohort (*n* = 2202). The area under the ROC curve was 0.708 (95% CI, 0.680–0.737; *P* < .001, Figure 1B), with a sensitivity of 50% and specificity of 79.2% at the cutoff of ≥ 3. This threshold yielded a PPV of 69.1%, NPV of 63.0%, LR^+^ of 2.40, and LR^−^ of 0.63, with a non-significant difference in AUC between derivation and validation cohorts (*P* = .454).

## Discussion

In this large, multicentre, observational study, nearly one in 2 patients experienced FR corroborating the findings from previous studies.^1^^,^^3^^,^^4^^,^^7^ The main findings of the current analysis are that FR is independently associated with older age, diabetes mellitus, ischaemic heart disease (IHD) and more severe stroke. Novel observations include the independent association of ACA occlusions and the presence of seizures on admission with FR. In contrast, administration of IVT prior to EVT appeared to be protective against FR, as did higher ASPECT/pcASPECT scores at baseline. Notably, hypertension, use of general anaesthesia, and time from symptom onset to treatment were not associated with FR in our analysis in contrast to previous studies.

Taken together, these insights may facilitate a more refined patient selection process and support better-informed decision-making, potentially helping to avoid interventions in individuals less likely to benefit from EVT.^8^^,^^9^^,^^11^^,^^19^

Previously proposed predictive tools for FR^8^^,^^12^^,^^13^ demonstrated strong statistical performance but their development was based on relatively small patient cohorts. Moreover, some excluded important radiological variables,^8^ while others relied on highly specific radiological findings.^12^ Most importantly, all of these models only included patients with anterior circulation LVO and excluded patients with posterior LVOs. These limitations restrict generalisability and reduce clinical applicability in broader stroke populations. In contrast, our analysis yielded an all-inclusive predictive score that integrates 8 readily available parameters, making it an easy-to-use score that is applicable to all LVO patients, irrespective of occlusion location.

In ROC curve analysis, a cutoff score of 3 was identified as optimal for predicting FR. Patients scoring < 3 had a high NPV (86.6%), while scores of 3–4 were defined as intermediate-risk category (PPV 68%–78%), and scores > 4 identified a distinctly high-risk group (PPV 87%). Our model demonstrated acceptable discrimination and high specificity, making it particularly effective for accurately identifying patients at high risk of FR. While its sensitivity was modest, this characteristic aligns with the intended purpose of the score which is not to serve as a broad screening tool for ruling out patients from treatment, but rather as a targeted risk-stratification instrument. Internal validation confirmed similar performance, underscoring the model’s robustness. We believe these findings support the use of FRET as a practical tool that can aid in early identification of patients unlikely to benefit from EVT, however, it should be noted that it is not perfect and therefore the decision of whether or not to proceed with EVT should be carefully weighed for each individual patient. On comparison of the FRET scores with previously published scores on the same sample of EVATRISP patients, the FRET score performed similarly. Nevertheless, we believe FRET has the advantages of being validated in a much larger sample and being designed to include all patients with LVO irrespective of the artery involved or core size.

The pathophysiology of FR is multifactorial and not fully understood. One proposed mechanism is the no-reflow phenomenon, characterised by persistent microvascular failure to reperfuse brain tissue despite successful recanalisation.^19^^,^^20^ Other mechanisms that could be responsible for FR may include distal embolisation to uninvolved territories and complications associated with EVT.

Both diabetes mellitus and ischaemic heart disease have been previously associated with FR.^11^^,^^19–21^ A plausible explanation for the link of FR with diabetes is chronic microvascular dysfunction which can impair neuronal recovery following acute ischaemia.^22^ Of note, stress hyperglycemia was also found to be associated with FR.^23^ However, since our sample did not include admission glucose evaluation in most patients this could not be assessed in the current analysis.

The current findings support the observation that advancing age is associated with a higher likelihood of poor outcomes. This raises important considerations regarding the appropriateness of intervention in elderly patients, particularly when multiple risk factors identified in our study are present.^24^^,^^25^ The higher likelihood of concomitant vascular disease such as IHD in the elderly may link age and FR via the mechanisms discussed above. Nevertheless, it is widely recognised that biological age is much more relevant than chronological age, and that some octogenarians and nonagenarians can achieve favourable outcomes following EVT.^26^ Therefore, patients should not be excluded from EVT solely on the basis of chronological age.

A high NIHSS score combined with a low ASPECTS at presentation have been identified in previous studies as independent predictors of FR.^8^^,^^10^^,^^11^ The strong association observed in our cohort likely reflects the presence of a large ischaemic burden, both clinically and radiologically, which limits the potential benefit of EVT.

Previous studies have shown an association between FR and the development of post-stroke epilepsy following EVT.^27^ In our cohort, an intriguing and unexpected finding was the association between epileptic seizures at presentation and subsequent FR. The underlying mechanism linking early seizures to FR remains unclear but may involve increased metabolic demand and accelerated enlargement of the ischaemic core. This also raises the possibility that shared pathological processes, could contribute both to seizure activity and to poor functional outcomes, warranting further investigation.^28^

Anterior cerebral artery occlusion was associated with FR in our cohort. This finding is supported by recent studies, and may be related to the technical complexity of EVT in these patients and potentially higher rates of procedural complications.^29^

Use of tPA was associated with a lower likelihood of FR corroborating prior findings and recommendations.^30^ This benefit may involve not only partial clot lysis but also effects at the microvascular level, which enhance reperfusion, and potentially reducing no-reflow phenomenon.

Futile recanalisation is associated with various post-procedural complications, including ICH, which negatively affect outcomes.^11^^,^^19^ These associations may be explained by technical complications during EVT which can prolong hospitalisation and worsen clinical outcomes.^31^ Another mechanism that could correlate with FR is vessel reocclusion, however we did not have data regarding this phenomenon.^32^

Key strengths of our study include its large, prospectively enrolled, and all-inclusive cohort, which enhances both statistical power and generalisability.

However, several limitations should be noted. First, missing data for a substantial proportion of patients in the EVATRISP cohort led to their exclusion from the analysis. Second, the retrospective and observational design may introduce inherent bias. Third, the absence of a centralised core imaging laboratory may have introduced variability in image interpretation, and certain potentially relevant variables. Fourth, while it may be contended that achieving a mRS ≤ 2 is an impractical therapeutic target in the majority of patients presenting with a large infarct core, and that a more lenient threshold such as mRS ≤ 3 or even 4 may be more appropriate for defining functional recovery in this subgroup, our cohort comprised a representative sample of patients with both large and small core infarcts. Our objective was to develop a clinically applicable scale suitable for use across the spectrum of LVO, irrespective of infarct territory or volume. Accordingly, we adopted the commonly utilised definition of FR as mRS > 2.^10^^,^^19^^,^^33^ Fifth, the clinical decision pathways to performing EVT as well as the technique used were not specified for each individual case. Thus, the changes in indications for EVT and technical improvements over the years could not be taken into account and we cannot exclude the possibility that this may have impacted the results. Another limitation pertains to the point that we did not have absolute TICI scores available to us and that precluded us from analysing the data according to TICI scores of 2b vs 2c vs 3. This is of importance because TICI2c and TICI 3 offer better chances of attaining functional independence at 90 days.^34^^,^^35^ Unfortunately, TICI data was coded in EVATRISP as successful (≥2b) or unsuccessful (≤2a) without mention of the absolute TICI value at the end of EVT. Finally, a key limitation of our predictive score is that it has undergone only internal validation, and its generalisability to other populations remains to be confirmed through external validation.

In conclusion, FR remains a prevalent outcome following EVT and is associated with increased rates of post-procedural complications and mortality. This study identified several independent predictors of FR. The FRET score is proposed as a pragmatic bedside instrument for early risk stratification in patients presenting with LVO, regardless of the affected vascular territory and may be used by clinicians at bedside and could easily be incorporated into the work flow to further inform family and caregivers regarding the likelihood of futile procedures. However, we believe that it should not be used to exclude patients from treatment. In cases where multiple high-risk parameters are present, the score may support re-evaluation of the indication for EVT to mitigate the likelihood of futile recanalisation and to enhance overall treatment efficiency.

## Supplementary Material

aakaf013_Supplementary_Figures_revision_1

aakaf013_Supplementary_Tables_Revision_1

## Data Availability

Data presented here can be made upon request to the PI.
