# Supplementary material for: FRET score: predictors of futile recanalisation following endovascular thrombectomy—a multicentre cohort study from the EVATRISP collaboration
Source: Eur Stroke J. 2026 Jan 1;11(1):aakaf013. doi: 10.1093/esj/aakaf013 (PMC12866636; doi:10.1093/esj/aakaf013)
Supplement: aakaf013_Supplementary_Figures_revision_1 [file aakaf013_supplementary_figures_revision_1.docx]

Supplementary Figure 1:


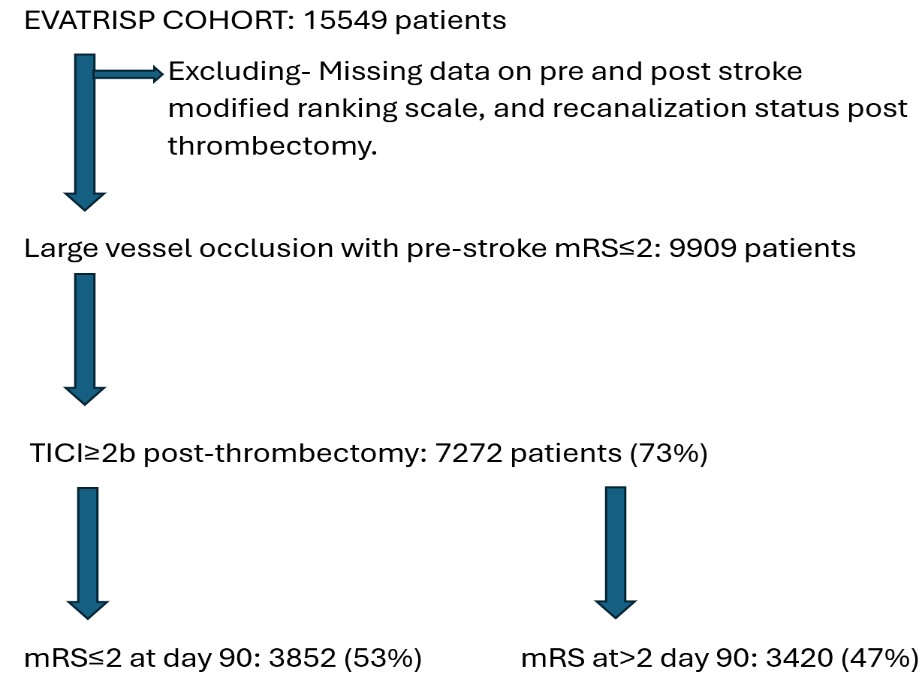


Supplementary Figure 2:


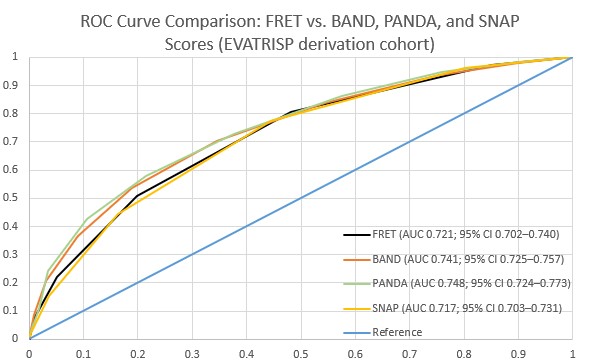


Supplementary Figure legends:

Supplementary Figure 1: Flow chart of patients included in the study

mRS: modified Rankin Scale; TICI: Thrombolysis in cerebral infarction

Supplementary Figure 2: Comparison of the FRET score to previously published scoring systems

AUC: area under the curve; ROC: receiver operator curve
