# Supplementary material for: FRET score: predictors of futile recanalisation following endovascular thrombectomy—a multicentre cohort study from the EVATRISP collaboration
Source: Eur Stroke J. 2026 Jan 1;11(1):aakaf013. doi: 10.1093/esj/aakaf013 (PMC12866636; doi:10.1093/esj/aakaf013)
Supplement: aakaf013_Supplementary_Tables_Revision_1 [file aakaf013_supplementary_tables_revision_1.docx]

Supplementary Table 1. Baseline characteristics of included vs. excluded patients

| Variable | Included patients (N=9909) | Excluded patients (N=5640) | p |
| --- | --- | --- | --- |
| Sex male (%) | 5271 (53.1) | 2793 (49.5) | <0.001 |
| Age (median, IQR) | 74 (63-82) | 76 (65-85) | <0.001 |
| Transferred from another hospital (%) | 3541 (35.7) | 2229 (39.5) | <0.001 |
| Atrial fibrillation (%) | 3499 (35.3) | 2033 (36.0) | 0.365 |
| Diabetes mellitus (%) | 1823 (18.4) | 1133 (20.1) | 0.011 |
| Hypertension (%) | 6657 (67.2) | 3695 (65.5) | 0.032 |
| Dyslipidemia (%) | 4775 (48.2) | 2500 (44.3) | <0.001 |
| Smoking (%) | 2098 (21.2) | 947 (16.8) | <0.001 |
| Coronary heart disease (%) | 1719 (17.3) | 976 (17.3) | 0.947 |
| Prior ischemic stroke (%) | 1182 (11.9) | 905 (16.0) | <0.001 |
| Wake up stroke (%) | 1606 (16.2) | 725 (12.9) | <0.001 |
| Epileptic seizure at presentation (%) | 97 (1.0) | 42 (0.7) | 0.157 |
| NIHSS score at presentation (median, IQR) | 14 (8-19) | 15 (9-20) | <0.001 |
| ASPECT/pcASPECTS score (median, IQR) | 9 (8-10) | 9 (8-10) | <0.001 |
| Symptoms onset to IVT, minutes (mean±SD) | 130±163 | 126±91 | 0.467 |
| Symptoms onset to groin puncture, minutes (mean±SD) | 276±401 | 268±395 | 0.432 |

ASPECTS: Alberta Stroke Program Early CT Score; IVT: intravenous thrombolysis; NIHSS: national institutes of health stroke scale;

Supplementary Table 2: Univariate analysis with futile recanalization defined as mRS>3

| Variable | mRS≤3 (N=3494) | mRS>3 (N=1580) | p |
| --- | --- | --- | --- |
| Sex male (%) | 1890 (54.1) | 792 (50.1) | 0.008 |
| Age (median, IQR) | 71 (61-79) | 78 (70-84) | <0.001 |
| Transferred from another hospital (%) | 1136 (32.5) | 570 (36.1) | 0.013 |
| Atrial fibrillation (%) | 1143 (32.7) | 679 (43.0) | <0.001 |
| Diabetes mellitus (%) | 570 (16.3) | 395 (25.0) | <0.001 |
| Hypertension (%) | 2257 (64.6) | 1201 (76.0) | <0.001 |
| Dyslipidaemia (%) | 1742 (49.9) | 794 (50.3) | 0.808 |
| Smoking (%) | 838 (24.0) | 244 (15.4) | <0.001 |
| Coronary heart disease (%) | 587 (16.8) | 332 (21.0) | <0.001 |
| Prior ischemic stroke (%) | 361 (10.3) | 234 (14.8) | <0.001 |
| Wake up stroke (%) | 540 (15.5) | 242 (15.3) | 0.441 |
| Epileptic seizure at presentation (%) | 26 (0.7) | 22 (1.4) | 0.023 |
| NIHSS score at presentation (median, IQR) | 12 (7-17) | 17 (13-21) | <0.001 |
| TOAST criteria  Large vessel arteriopathy (%)  Cardiac source (%)  More than one etiology (%)  Undetermined (%)  Other (%) | 20.4  44.5  5.6  21.8  7.8 | 20.1  47.9  6.2  19.1  7.2 | 0.131 |
| ASPECTS score (median, IQR) (*n=2863) | 9 (8-10) | 8 (7-10) | <0.001 |
| Early ischemic changes on computed tomography (%) | 1046 (29.9) | 556 (35.2) | <0.001 |
| TAN score in M1/2 occlusion (median, IQR) (*n= 1058) | 2 (1-2) | 1 (1-2) | <0.001 |
| Vessel occlusion  Internal carotid (%)  Terminus carotid (%)  Middle cerebral artery proximal M1 (%)  Middle cerebral artery distal M1 (%)  Middle cerebral artery M2 (%)  Anterior cerebral artery (%)  Posterior cerebral artery (%)  Basilar artery (%)  Vertebral artery (%) | 235 (6.7)  380 (10.9)  1133 (32.4)  538 (15.4)  830 (23.8)  42 (1.2)  123 (3.5)  194 (5.6)  42 (1.2) | 114 (7.2)  279 (17.7)  552 (34.9)  221 (14.0)  281 (17.8)  36 (2.3)  41 (2.6)  121 (7.7)  37 (1.7) | 0.549  0.001  0.082  0.202  <0.001  0.004  0.087  0.004  0.149 |

ASPECTS: Alberta Stroke Project Early CT Score; mRS: modified Rankin Scale; NIHSS: National Institutes of Health Stroke Scale; TOAST: Trial of ORG 10172 in Acute Stroke Treatment

Supplementary Table 3: Multivariate analysis of futile recanalization according to mRS>3

|  | Adjusted OR | 95% CI | p |
| --- | --- | --- | --- |
| Sex male | 1.02 | 0.85-1.23 | 0.792 |
| Age | 1.04 | 1.03-1.06 | <0.001 |
| Transferred from another hospital | 1.10 | 0.89-1.35 | 0.363 |
| Atrial fibrillation | 0.96 | 0.79-1.17 | 0.695 |
| Diabetes mellitus | 1.63 | 1.32-2.02 | <0.001 |
| Hypertension | 1.27 | 1.02-1.59 | 0.034 |
| Smoking | 0.98 | 0.77-1.25 | 0.883 |
| Coronary heart disease | 1.08 | 0.86-1.36 | 0.488 |
| Prior ischemic stroke | 1.32 | 1.01-1.73 | 0.046 |
| Seizure at stroke | 1.84 | 0.74-4.57 | 0.187 |
| NIHSS at admission | 1.09 | 1.08-1.11 | <0.001 |
| Tissue plasminogen activator | 0.55 | 0.45-0.66 | <0.001 |
| ASPECT/pcASPECTS score | 0.81 | 0.77-0.86 | <0.001 |
| Early ischemic changes on Computed tomography | 0.98 | 0.81-1.19 | 0.878 |
| **Vessel involved**  Terminus carotid  Middle cerebral artery M2  Anterior cerebral artery  Basilar artery | 1.69  0.84  1.68  1.41 | 1.32-2.19  0.69-1.05  0.92-3.07  0.77-2.54 | <0.001  0.125  0.089  0.259 |

ASPECTS: Alberta Stroke Project Early CT Score; CI: confidence intervals; NIHSS: National Institutes of Health Stroke Scale; OR: odds ratio

Supplementary Table 4: Regression analysis based on previously published factors know to be associated with outcomes:

| Variable | Adjusted OR | 95% CI | p |
| --- | --- | --- | --- |
| Age | 1.044 | 1.034-1.054 | <0.001 |
| Pre-Stroke mRS | 1.611 | 1.368-1.898 | <0.001 |
| NIHSS score | 1.103 | 1.084-1.122 | <0.001 |
| Time from symptoms onset to groin puncture | 1.001 | 1.000-1.001 | 0.001 |
| ASPECTS | 0.846 | 0.792-0.904 | <0.001 |

ASPECTS: Alberta Stroke Project Early CT Score; CI: confidence intervals; mRS: modified Rankin Scale; NIHSS: National Institutes of Health Stroke Scale; OR: odds ratio
